# The S-Transform of Distributions

**DOI:** 10.1155/2014/623294

**Published:** 2014-01-02

**Authors:** Sunil Kumar Singh

**Affiliations:** Department of Mathematics, Rajiv Gandhi University, Doimukh, Arunachal Pradesh 791112, India

## Abstract

Parseval's formula and inversion formula for the S-transform are given. A relation between the S-transform and pseudodifferential operators is obtained. The S-transform is studied on the spaces *𝒮*(ℝ^*n*^) and *𝒮*′(ℝ^*n*^).

## 1. Introduction

The S-transform was first used by Stockwell et al. [1] in 1996. If *ω*(*t*, *ξ*) is a window function, then the continuous S-transform of *ϕ*(*t*) with respect to *ω* is defined as [2]

(1) (Sωϕ)(τ,ξ)=∫ℝnϕ(t)ω(τ−t,ξ)e−i2π〈t,ξ〉dt,           for  x,ξ∈ℝn.

In signal analysis, at least in dimension *n* = 1, ℝ^2*n*^ is called the time-frequency plane, and in physics ℝ^2*n*^ is called the phase space.

Equation (1) can be rewritten as a convolution as

(2) $$\begin{array}{c} \left({\text{S}}_{\omega }\phi \right)\left(\tau ,\xi \right)=\left(\phi \left(\cdot \right)e^{-i2\pi \langle \cdot ,\xi \rangle }*\omega \left(\cdot ,\xi \right)\right)\left(\tau \right). \end{array}$$

Now, we recall the definitions of Fourier transform on ℝ^*n*^.

**Definition 1**

If *ϕ*(*t*) is defined on ℝ^*n*^, then the Fourier transform of *ϕ* is given by

(3) $$\begin{array}{c} ℱ\left[\phi \left(t\right)\right]\left(\xi \right)=\int_{{\mathbb{R}}^{n}}\phi \left(t\right)e^{-i2\pi \langle t,\xi \rangle }dt, \end{array}$$

where 〈*t*, *ξ*〉 = ∑_*j*=1_^*n*^ *t*_*j*_ *ξ*_*j*_ is the usual inner product on ℝ^*n*^.

**Definition 2**

If *ϕ*(*t*, *x*) is defined on ℝ^2*n*^, then the partial Fourier transform of *ϕ*(*t*, *x*) with respect to the first coordinate is given by

(4) $$\begin{array}{c} {ℱ}_{1}\left[\phi \left(t,x\right)\right]\left(\xi ,x\right)=\int_{{\mathbb{R}}^{n}}\phi \left(t,x\right)e^{-i2\pi \langle t,\xi \rangle }dt, \end{array}$$

and the partial Fourier transform of *ϕ*(*t*, *x*) with respect to the second coordinate is given by

(5) $$\begin{array}{c} {ℱ}_{2}\left[\phi \left(t,x\right)\right]\left(t,\eta \right)=\int_{{\mathbb{R}}^{n}}\phi \left(t,x\right)e^{-i2\pi \langle x,\eta \rangle }dx. \end{array}$$

Applying the convolution property for the Fourier transform in (2), we obtain

(6) $$\begin{array}{c} \left({\text{S}}_{\omega }\phi \right)\left(\tau ,\xi \right)={ℱ}_{1}^{-1}\left[{ℱ}_{1}\left(\phi \right)\left(\alpha +\xi \right){ℱ}_{1}\left(\omega \right)\left(\alpha ,\xi \right)\right]\left(\tau ,\xi \right), \end{array}$$

where *ℱ*_1_^−1^ is the inverse Fourier transform.

Now, we define the translation, modulation, and involution operators, respectively, by

(7) Tτϕ(t)=ϕ(t−τ)  (translation)Mξϕ(t)=ei2π〈ξ,t〉ϕ(t)  (modulation)ℐϕ(t)=ϕ(−t)¯  (involution),

where *t*, *τ*, *ξ* ∈ ℝ^*n*^.

**Definition 3 (the Dirac delta)**

The Dirac delta function is defined by

(8) $$\begin{array}{c} \iint_{{\mathbb{R}}^{n}}^{}\phi \left(t,x\right)\delta \left(t,x\right)dt\;dx=\phi \left(0,0\right). \end{array}$$

**Definition 4 (tempered distribution)**

A function *ϕ* ∈ *C* ^*∞*^(ℝ^*n*^) is said to be rapidly decreasing if

(9) $$\begin{array}{c} \gamma_{\alpha ,\beta }\left(\phi \right)=\underset{x\in {\mathbb{R}}^{n}}{\sup}\left|x^{\alpha }D^{\beta }\phi \left(x\right)\right|<\infty , \end{array}$$

for all pairs of multi-indices *α*, *β* ∈ *ℕ*_0_^*n*^. The space of all rapidly decreasing functions on ℝ^*n*^ is denoted by *𝒮*(ℝ^*n*^) or simply *𝒮*. Elements in the dual space *𝒮*′ of *𝒮* are called tempered distribution.

## 2. Some Important Properties of S-Transform

Some properties of S-transform can be found in [3–8] and certain properties of S-transform are obtained in this section. By definition, we have

(10) (Sωϕ)(τ,ξ)=∫ℝnω(τ−t,ξ)ϕ(t)e−i2π〈t,ξ〉dt=∫ℝnω(−x,ξ)ϕ(τ+x)e−i2π〈τ+x,ξ〉dx=∫ℝnω(−x,ξ)M−ξϕ(τ+x)dx=∫ℝnω(−x,ξ)T−τM−ξϕ(x)dx.

Thus, the S-transform S_*ω*_ appears as a superposition of time-frequency shifts as follows:

(11) $$\begin{array}{c} {\text{S}}_{\omega }:=\int_{{\mathbb{R}}^{n}}\omega \left(-x,\xi \right)T_{-\tau }M_{-\xi }dx. \end{array}$$

**Example 5**

If *ω*(*t*, *ξ*) = *m*(*ξ*), that is, independent of *t*, then

(12) (Sωϕ)(τ,ξ)=∫ℝnm(ξ)ϕ(t)e−i2π〈t,ξ〉dt=m(ξ)(ℱϕ)(ξ).

So S_*ω*_ is a multiplication operator. In particular, if *ω*(*t*, *ξ*) = 1, then (S_*ω*_ *ϕ*)(*τ*, *ξ*) = (*ℱϕ*)(*ξ*).

**Example 6**

If *ω*(*t*, *ξ*) = *m*(*t*), then

(13) (Sωϕ)(τ,ξ)=∫ℝnm(τ−t)ϕ(t)e−i2π〈t,ξ〉dt=∫ℝnT−τm(−t)ϕ(t)e−i2π〈t,ξ〉dt=∫ℝnT−τℐm¯(t)ϕ(t)e−i2π〈t,ξ〉dt=ℱ(ϕT−τℐm¯)(ξ).

**Theorem 7 (Parseval's formula)**

Let *ω*_1_ and *ω*_2_ be the window functions such that

(14) $$\begin{array}{c} \int_{{\mathbb{R}}^{n}}\omega_{1}\left(\tau -t,\xi \right)\bar{\omega_{2}\left(\tau -x,\eta \right)}\;d\tau =\delta \left(x-t,\xi -\eta \right). \end{array}$$

Let *ϕ*_1_, *ϕ*_2_ ∈ *L* ^2^(ℝ^*n*^) and let (*S*_*ω*_1__ *ϕ*_1_) and (*S*_*ω*_2__ *ϕ*_2_) be the *S*-transforms of *ϕ*_1_ and *ϕ*_2_, respectively. Then

(15) ∬ℝn(Sω1ϕ1)(τ,ξ)(Sω2ϕ2)(τ,ξ)¯dτ dξ =∫ℝnϕ1(t)ϕ2(t)¯ dt.

This immediately implies the Plancherel formula

(16) $$\begin{array}{c} {||S_{\omega }\phi ||}_{L^{2}({\mathbb{R}}^{n}\times {\mathbb{R}}^{n})}={||\phi ||}_{L^{2}({\mathbb{R}}^{n})}. \end{array}$$

**Proof**

Consider

(17) ∬ℝn(Sω1ϕ1)(τ,ξ)(Sω2ϕ2)(τ,ξ)¯ dτ dξ =∬ℝn(∫ℝnϕ1(t)ω1(τ−t,ξ)e−i2π〈t,ξ〉dt)    ×(∫ℝnϕ2(x)ω2(τ−x,ξ)e−i2π〈x,ξ〉dx)¯ dτ dξ =∭ℝnϕ1(t)ϕ2¯(x)ei2π〈(x−t),ξ〉    ×(∫ℝnω1(τ−t,ξ)ω2(τ−x,ξ)¯dτ)dξ dt dx =∭ℝnϕ1(t)ϕ2¯(x)ei2π〈(x−t),ξ〉δ(x−t,0)dξ dt dx =∫ℝnϕ1(t)ϕ2(t)¯dt.

**Theorem 8 (inversion formula)**

If *ϕ* ∈ *L* ^2^(ℝ^*n*^) and window function *ω* satisfy the condition (14) of the previous theorem, then

(18) $$\begin{array}{c} \phi \left(t\right)=\iint_{{\mathbb{R}}^{n}}^{}\left(S_{\omega }\phi \right)\left(\tau ,\xi \right)\bar{\omega \left(\tau -t,\xi \right)}e^{i2\pi \langle t,\xi \rangle }d\tau \;d\xi . \end{array}$$

**Proof**

By the previous theorem we can write

(19) 〈ϕ1,ϕ2〉=∬ℝn(Sωϕ1)(τ,ξ)(Sωϕ2)(τ,ξ)¯ dτ dξ=∬ℝn(Sωϕ1)(τ,ξ)   ×(∫ℝnϕ2(x)ω(τ−x,ξ)e−i2π〈x,ξ〉dx)¯ dτ dξ=∫ℝn(∬ℝn(Sωϕ1)(τ,ξ)      × ω(τ−x,ξ)¯ei2π〈x,ξ〉dτ dξ)ϕ2¯(x)dx.

Hence

(20) $$\begin{array}{c} \phi_{1}\left(t\right)=\iint_{{\mathbb{R}}^{n}}^{}\left({\text{S}}_{\omega }\phi_{1}\right)\left(\tau ,\xi \right)\bar{\omega \left(\tau -t,\xi \right)}e^{i2\pi \langle t,\xi \rangle }d\tau \;d\xi . \end{array}$$

**Definition 9**

Let *ω* be a window function and S_*ω*_ is the S-transform. Then the transform S_ω_* defined by

(21) $$\begin{array}{c} \langle {\text{S}}_{\omega }\phi ,\psi \rangle =\langle \phi ,{\text{S}}_{\omega }^{*}\psi \rangle \end{array}$$

is called the adjoint of S_*ω*_. If *ϕ* ∈ *L* ^2^(ℝ^*n*^) and *ψ* ∈ *L* ^2^(ℝ^*n*^ × ℝ^*n*^), then (21) implies that

(22) $$\begin{array}{c} \left({\text{S}}_{\omega }^{*}\psi \right)\left(t\right)=\iint_{{\mathbb{R}}^{n}}^{}\psi \left(\tau ,\xi \right)\bar{\omega \left(\tau -t,\xi \right)}e^{i2\pi \langle t,\xi \rangle }d\tau \;d\xi , \end{array}$$

where *t*, *τ*, *ξ* ∈ ℝ^*n*^.

Theorem 10 (Parseval's formula for S_ω_*)

Let *ω*_1_ and *ω*_2_ be the window functions that satisfy the condition (14). If *ψ*_1_, *ψ*_2_ ∈ *L* ^2^(ℝ^*n*^ × ℝ^*n*^), then

(23) ∫ℝn(Sω1∗ψ1)(t)(Sω2∗ψ2)(t)¯ dt  =∬ℝnψ1(τ,ξ)ψ2(τ,ξ)¯ dτ dξ,

and the Plancherel formula is

(24) $$\begin{array}{c} {||S_{\omega }^{*}\psi ||}_{L^{2}({\mathbb{R}}^{n})}={||\psi ||}_{L^{2}({\mathbb{R}}^{n}\times {\mathbb{R}}^{n})}. \end{array}$$

**Proof**

Consider

(25) ∫ℝn(Sω1∗ψ1)(t)(Sω2∗ψ2)(t)¯ dt=∫ℝn(∬ℝnψ1(τ,ξ)ω1(τ−t,ξ)¯ei2π〈t,ξ〉dτ dξ)   ×(∬ℝnψ2(α,η)ω2(α−t,η)¯ei2π〈t,η〉dα dη)¯ dt=∫∭ℝnψ1(τ,ξ)ψ2(α,η)¯ei2π〈t,ξ−η〉     ×(∫ℝnω1(τ−t,ξ)¯ω2(α−t,η)dt)dτ dξ dα dη=∫∭ℝnψ1(τ,ξ)ψ2(α,η)¯ei2π〈t,ξ−η〉     ×δ(τ−α,ξ−η)dτ dξ dα dη=∬ℝnψ1(τ,ξ)ψ2(τ,ξ)¯ dτ dξ.

This proves the theorem.

**Theorem 11**

If the window function *ω* satisfies the condition (14), then

(26) $$\begin{array}{c} S_{\omega }S_{\omega }^{*}=I=S_{\omega }^{*}S_{\omega }, \end{array}$$

where *I* is the identity operator.

**Proof**

By definition

(27) Sω∗[Sω1ψ](t)=∬ℝn(Sω1ψ)(τ,ξ)ω(τ−t,ξ)¯ei2π〈t,ξ〉dτ dξ=Sω−1[Sω1ψ](t).

Thus

(28) $$\begin{array}{c} {\text{S}}_{\omega }^{*}={\text{S}}_{\omega }^{-1}. \end{array}$$

This proves the theorem.

**Definition 12 (pseudodifferential operator)**

Let *σ* be a (measurable) function or a tempered distribution on ℝ^*n*^. Then the operator

(29) $$\begin{array}{c} K_{\sigma }\phi \left(t\right)=\int_{{\mathbb{R}}^{n}}\sigma \left(t,\xi \right)\hat{\phi }\left(\xi \right)e^{i2\pi \langle t,\xi \rangle }d\xi \end{array}$$

is called the pseudodifferential operator.

The pseudodifferential operator plays an important role in the theory of partial differential equations. The pseudodifferential operator has been studied on function and distribution spaces by many authors. Details of the concept can be found in [9, 10].

### 2.1. Relation between the S-Transform and Pseudodifferential Operator

Here we give a direct relation between S-transform and pseudodifferential operator which will may be very useful in the study of S-transform of distribution spaces. The continuous S-transform of a function *ϕ* with respect to a window function *ω* is given by

(30) (Sωϕ)(τ,ξ)=∫ℝnϕ(t)ω(τ−t,ξ)e−i2π〈t,ξ〉dt=∫ℝnϕ(τ−x)ω(x,ξ)e−i2π〈(τ−x),ξ〉dx=e−i2π〈τ,ξ〉∫ℝnT−τϕ(−x)ω(x,ξ)ei2π〈x,ξ〉dx=e−i2π〈τ,ξ〉∫ℝnℱ[ℱ(T−τϕ)](x)      ×ω(x,ξ)ei2π〈x,ξ〉dx=e−i2π〈τ,ξ〉Kσ[ℱ(T−τϕ)](ξ),

where *σ*(*ξ*, *x*) = *ω*(*x*, *ξ*).

## 3. The S-Transform of Distributions

In this section we will investigate the S-transform of tempered distribution by means of the Fourier transform.

**Theorem 13**

If *ω* ∈ *𝒮*(ℝ^2*n*^), then *S*_*ω*_ maps *𝒮*(ℝ^*n*^) into *𝒮*(ℝ^2*n*^).

**Proof**

By (6) we have

(31) $$\begin{array}{c} \left({\text{S}}_{\omega }\phi \right)\left(\tau ,\xi \right)={ℱ}_{1}^{-1}\left[{ℱ}_{1}\left(\phi \right)\left(\alpha +\xi \right){ℱ}_{1}\left(\omega \right)\left(\alpha ,\xi \right)\right]\left(\tau ,\xi \right). \end{array}$$

Thus, (S_*ω*_ *ϕ*) ∈ *𝒮*(ℝ^2*n*^), since the Fourier transform is continuous isomorphism from *𝒮*(ℝ^*n*^) to *𝒮*(ℝ^*n*^), and its inverse is also a continuous isomorphism from *𝒮*(ℝ^*n*^) to *𝒮*(ℝ^*n*^) (see [11], page 66-67).

**Theorem 14**

If *ω* ∈ *𝒮*(ℝ^2*n*^), then *S*_*ω*_ maps *𝒮*′(ℝ^*n*^) into *𝒮*′(ℝ^2*n*^).

**Proof**

For any *f* ∈ *𝒮*′(ℝ^*n*^) and *ψ* ∈ *𝒮*(ℝ^2*n*^), we have

(32) 〈(Sωf)(τ,ξ),ψ(τ,ξ)〉 =∬ℝn(∫ℝnf(t)ω(τ−t,ξ)e−i2π〈t,ξ〉dt)ψ(τ,ξ)¯ dτ dξ            (in  fact  ψ−=ψ, since  ψ∈𝒮) =∫ℝnf(t)(∬ℝnψ(τ,ξ)¯ω(τ−t,ξ)e−i2π〈t,ξ〉dτ dξ)dt =〈f,ϕ〉,

where

(33) ϕ(t)=∬ℝnψ(τ,ξ)ω(τ−t,ξ)¯ei2π〈t,ξ〉dτ dξ=∫ℝn(ψ∗(ℐω))(t,ξ)ei2π〈t,ξ〉dξ=ℱ2−1(ψ∗(ℐω))(t)∈𝒮(ℝn).

Thus, (S_*ω*_ *f*)(*τ*, *ξ*) ∈ *𝒮*′(ℝ^2*n*^).

**Theorem 15**

If *ω* ∈ *𝒮*′(ℝ^2*n*^), then *S*_*ω*_ maps *𝒮*(ℝ^*n*^) into *𝒮*′(ℝ^2*n*^).

**Proof**

If *ϕ* ∈ *𝒮*(ℝ^*n*^) and *ψ* ∈ *𝒮*(ℝ^2*n*^), then

(34) $$\begin{array}{c} U_{\phi ,\psi }\left(\tau ,\xi \right):=\bar{ℱ\left(\phi \right)\left(\alpha +\xi \right)}\psi \left(\alpha ,\xi \right)\in 𝒮\left({\mathbb{R}}^{2n}\right). \end{array}$$

Thus for any *ω* ∈ *𝒮*′(ℝ^2*n*^), we have

(35) 〈(ℱ1ω)(α,ξ),Uϕ,ψ(α,ξ)〉 =∬ℝn(ℱ1ω)(α,ξ)(ℱϕ)(α+ξ)ψ(α,ξ)¯ dα dξ =∬ℝn(ℱ1(Sωϕ))(α,ξ)ψ(α,ξ)¯ dα dξ =〈(ℱ1(Sωϕ))(α,ξ),ψ(α,ξ)〉.

Thus *ℱ*_1_(S_*ω*_ *ϕ*) ∈ *𝒮*′(ℝ^2*n*^) and hence (S_*ω*_ *ϕ*) ∈ *𝒮*′(ℝ^2*n*^).
